# The Meaning of Research

**Published:** 1919-12-27

**Authors:** 


### THE MEANING OF RESEARCH.

It comes as a logical sequence that, after a period of active propaganda to ensure the institution, or more correctly the adequate continuation, of medical research on national lines, we should arrive at a phase of universal discussion, not only as to how best to bring this about, but also as to exactly and precisely what it is that we are trying to institute. The simplicity and conciseness of the term "medical research" corresponds apparently in an inverse ratio to that of the issues actually involved, and although it is an undoubted fact that if we have the will and enterprise, we may now inaugurate a new epoch in the medical history of this country and ultimately place on record many triumphs for British brains and effort, yet we must not fall into the error of supposing the paths ahead to be clearly defined.

Thus the number of pronouncements which have recently appeared both directly from the pens of eminent scientists and in the pages of our more reliable lay contemporaries, are most valuable in throwing a clearer light on the requirements of the problem which the country has rightly set itself to solve. Also a most important development occurs with the freeing of the Medical Research Committee. The formal development which this body, with its admirable war-time record, is undergoing should add immensely to its prestige, its resources, and its services to the nation. The National Health Insurance Commission, of which the Research Committee was an appendage, is to be no more, but by a provision which has caused no little misunderstanding, the research unit is not automatically transferred to the new Ministry of Health. As we have seen it most aptly explained, the arrangement now afoot is not an oversight but an instance of rare foresight. Those responsible acted upon the practical realisation that medical research, in the true sense of the term and quite distinct from the routine laboratory investigation of all manner of specimens from the sick man—a most necessary provision but decidedly *not* research—is too big a thing to be carried on in any petty, semi-co-ordinated fashion under a number of separate committees in a number of different centres. It is absolutely essential to have a controlling body of the highest status, of the widest outlook and powers, and fortunately for the successful accomplishment of the many tasks ahead, the country will now have a research committee cramped under the wing of no controlling department, and freed from the liability to confinement to a purely medical outlook; the two greatest hindrances in the past. Actually there is no such thing as "medical research," nor yet as "medical science." But there is research and there is science; and medicine, the art of healing, consists of the application to the needs of mankind of all and every part of science which will serve to improve or benefit his health.

For these permanent reasons the decision has been made to transfer the Medical Research Committee to the Privy Council so that it shall be immune from imprisonment by either territorial barriers or by irritating obstruction, arising with unavoidable limitations of the purely administrative mind with its invariable and most destructive tendency to dissect knowledge of the universe into this and that "science," each carefully defined and isolated from the rest, and all, therefore, in a starving and moribund condition. It was the chemist who gave us salvarsan. Pasteur was a chemist and, strictly, it is to biologists and entomologists that we owe no small proportion of our most recent medical advances. What the country needs is research in the widest sense, and national support and encouragement towards its accomplishment, and there must be public as well as the recent official appreciation that, though health problems are primarily concerned, the medical profession and the hospitals represent but a tithe of the scientific units involved.

